# Attempted suicide short intervention program for older adults 65+ (ASSIP-OA): a study protocol for a multicentre randomised controlled trial

**DOI:** 10.1186/s12888-025-07016-7

**Published:** 2025-06-06

**Authors:** Sara Hed, Anne Ingeborg Berg, Maria Tillfors, Anna Ehnvall, Åsa Westrin, Stefan Wiktorsson, Anja Gysin-Maillart, Margda Waern

**Affiliations:** 1 Department of Psychiatry and Neurochemistry, Institute of Neuroscience and Physiology, University of Gothenburg, Blå Stråket 15, SU/Sahlgrenska, Gothenburg, 413 45 Sweden; 2 Department of Neuropsychiatry, Sahlgrenska University Hospital, Region Västra Götaland, Gothenburg, Sweden; 3 Department of Psychology, University of Gothenburg, Gothenburg, Sweden; 4 Department of Social and Psychological Sciences, Karlstad University, Karlstad, Sweden; 5 Psychiatric Outpatient Clinic, Region Halland, Varberg, Sweden; 6 Department of Psychiatry, Skåne University Hospital, Lund, Sweden; 7 Unit for Clinical Suicide Research, Department of Clinical Sciences, Faculty of Medicine, Lund University, Lund, Psychiatry Sweden; 8 Translational Research Center, University Hospital of Psychiatry, University of Bern, Bern, Switzerland

**Keywords:** Older adults, Suicide attempt, Suicide preventions, Multicentre randomised controlled trial, Attempted suicide short intervention program

## Abstract

**Background:**

Older adults are underrepresented in suicide prevention research, even though suicide rates are higher in this age group (65+) than in any other age group in many countries worldwide. There are few clinical intervention studies that target this age group. A promising preventive intervention is the Attempted Suicide Short Intervention Program (ASSIP). Given differences in suicidal behaviour between older and younger adults, there is a need for suicide prevention intervention models that are adaptable to the heterogenic needs of older adults. This study protocol outlines the design of our upcoming study, which aims to evaluate a modified version of the Attempted Suicide Short Intervention Program (ASSIP) specifically adapted for older adults (65+), ASSIP for Older Adults (ASSIP-OA), as add-on to Treatment as Usual (TAU) and compared to TAU only.

**Methods:**

A multicentre two-group parallel randomised controlled trial will be conducted to compare TAU to ASSIP-OA (3–5 sessions) and standardized letters for 2 years as an add-on to treatment as usual. The trial, which is open labelled, will recruit 132 participants (65+) within psychiatric services in Sweden after a suicide attempt or hospitalisation for serious suicidal plans. The main modifications in comparison with the original ASSIP include (a) flexibility in treatment delivery (offering home visits, breaks and shorter/longer sessions as needed), (b) an additional session together with relatives or other support person(s), and (c) age-specific linguistic and content-wise adaptation of homework, letters, and case conceptualization. The primary outcome is a suicidal episode (fatal or non-fatal). Secondary outcomes include psychiatric symptoms, severity of suicidal ideation, coping style and quality of life. The trial also includes measures of feasibility, health-care utilization and negative effects of treatment.

**Discussion:**

This study has the potential to inform the development and implementation of a more person-centred care for suicidal older adults.

**Trial registration:**

ClinicalTrials.gov: NCT06831942. Registered February 21, 2025, revised May 21, 2025.

**Supplementary Information:**

The online version contains supplementary material available at 10.1186/s12888-025-07016-7.

## Background

Older adults are sorely underrepresented in suicide prevention research, despite the fact that suicide rates are higher in this age group (65+) than in any other age group in Sweden [1] and in many western countries worldwide [2]. There are few clinical preventive intervention studies that target this age group [3], and to the best of our knowledge there are to date no published studies set in Europe.

Older adult suicidal behaviour is a phenomenon involving complex processes of human behaviour that evolve over time, influenced by numerous factors, as opposed to a “normal” response to the aging process [4]. According to the Interpersonal Theory of Suicide in Older Adults [5] social disconnectedness is a main catalyst for the development of late-life suicidal ideation and behaviours. This concept includes feelings of burdensomeness, as well as thwarted belongingness. An effective model for suicide prevention in older adults must therefore go beyond the medical model which tends to see suicidal ideation and behaviour as symptoms of depression. A more systemic model is necessary to shift the focus to include preventive measures that can be taken by the suicidal individuals themselves, as well as other sources of potential support, including professional caregivers. An interview study conducted by our research group with older adults in geriatric psychiatry following a suicide attempt revealed that many participants had a limited understanding of their suicidal behaviour [6]. Furthermore, the results suggests that this lack of comprehension itself may pose a risk for future suicidal behaviour. A preventive intervention aimed at constructing a narrative around the suicidal phase could enhance understanding and help individuals regain a sense of control and agency.

In Sweden today, antidepressants and other types of psychoactive medication constitute the main treatment strategy available for older adults with depression and suicidal issues. While evidence-based therapies are available for younger adults with mental health issues, this is not the case for the oldest members of the population, despite the fact that their need for specialised services is at least as great [7]. In our recent interview study [6], older adult suicide attempters shared that age-related physical decline increased their dependence on the healthcare system. However, their psychological distress and life-weariness were often met with a lack of response from both primary care doctors and specialists. Some reported that their experiences with the healthcare system deepened their hopelessness, leading them to believe no help was available. In line with our focus group study with older adults with depression [8] older adults described frustration about the healthcare they received. They wanted to talk with professional caregivers about their depressions, about their suicidal feelings and their existential issues, and about what they themselves could do to improve their mental health. These experiences, taken together with results showing that many older adults attempt [9] and die by suicide [10] despite antidepressant treatment, speak to the need for evidence-based psychosocial interventions for older adults who are depressed and suicidal. The authors of a recent review on psychosocial interventions for suicidal older adults concluded that the international literature was so scanty that no conclusions could be drawn [11].

Suicidal behaviour in older adults differs from that in younger adults in several ways. While suicidal older adults tend to score lower on symptom rating scales than younger persons, their suicidal behaviour is characterised by greater intent and higher level of planning compared to younger adults [12]. Thus, many older adults die at their first suicide attempt [13]. A prevention program for older adults can therefore not be limited to actual suicide attempts, but rather must also target older adults with serious suicidal plans. In addition to mental ill health issues (often depression, alcohol use disorders or a combination), suicidality in older adults is more often associated with comorbid physical illness, functional limitations, and milder forms of cognitive dysfunction compared to their younger counterparts. Compromised executive function [14] and limited capacity for problem solving [15] have been observed in research involving suicidal older adults. Cognition is affected also in normal ageing, including the capacity to solve new problems and to reflect on new situations [16]. This includes those with mild cognitive impairment, who may require additional support when coping with age-related challenges. Given the differences in suicidal behaviour between older and younger adults, there is a need for suicide prevention intervention models that can be adapted to address the heterogenic needs of older adults.

A promising preventive intervention is the Attempted Suicide Short Intervention Program (ASSIP) [17]. The ASSIP model is based on the premise that individual behaviour is understandable through the lens of the person’s life history, individual vulnerability, life goals, and basic needs. The original ASSIP was developed in Switzerland where it has shown, as add-on to Treatment As Usual (TAU), an 80% decrease in risk of new suicide attempts within 24 months compared to ordinary treatment in a clinical cohort of adults (mean age 38 years) [17]. The ASSIP study is one of 53 studies included in a preprint meta-analysis on brief interventions following a suicide attempt [18]. The meta-analysis found evidence that brief interventions reduced the risk of suicide re-attempts compared to any control condition. In Sweden, ASSIP is currently being tested for individuals aged 18 and older [19], and early data are encouraging [20].

ASSIP is a short, structured clinical intervention in which the therapist and the patient collaborate to develop strategies that increase patient empowerment and thereby strengthen the patient’s ability to cope with future suicidal crises. The ASSIP model is mainly based on action theory, attachment theory and cognitive theory of suicidal behaviour, in which suicide is seen as a coping strategy (albeit dysfunctional) that a person may opt to use in an unbearable situation of mental pain, where no other coping strategies appear available [21]. Suicidal behaviour is thus seen as “goal-directed”, the goal being to end mental pain [22]. The positive results of the Swiss ASSIP trial [17], appears to at least in part be related to improved coping strategies. Reduced dysfunctional coping, as well as greater problem- focused coping were observed in participants who took part in the ASSIP intervention. Further, ASSIP participants score higher on self-distraction (12 months) and lower on self-blame (24 months) compared to participants who received ordinary treatment [23]. The collaborative approach in ASSIP, the direct focus on suicidal behaviour, and the emphasis on developing strategies to enhance patient empowerment and coping skills from a lifespan perspective appears to align with the needs expressed by older adults [6, 24]. However, further emphasis on engaging social support including support from professional caregivers [25], may be beneficial in counteracting social disconnectedness, a salient feature of suicidal behaviour in older age groups [26, 27].

In its original form, ASSIP is a 3–4 session intervention, an add-on to usual treatment, which is initiated shortly after a suicide attempt [21]. The ASSIP therapist encourages patient to share their own personal history, putting a spotlight on the patient’s narrative regarding their suicide attempt, including events and emotions. This first session is video-recorded and played back at the second session, as the patient, and the therapist sit side-by-side and watch the film, identifying triggers, warning signs, and the participant’s suicidal mode including automatic thoughts, emotions and bodily reactions that were experienced during the suicidal crisis. This can be used to replace dysfunctional coping strategies with more functional ones, to hinder the development of the suicidal mode in the future. The aim is the identification of biographical vulnerabilities associated with suicidal behaviour, and to jointly develop long-term therapy goals that need to be shared with the longer-term health care network (e.g. psychotherapists, mental health professionals, GPs, caregivers, etc.). Homework is completed after the first session. It is psychoeducational in nature; participants make notes to ensure that the description of events in the homework text is in line with their own experiences. At the third session, the patient and the therapist revise the patients personal case conceptualisation of the suicidal behaviour, a summary of biographical vulnerabilities, individual triggering events, and suicidal process, as well as relevant therapy goals and warning signs (long-term and acute). Safety strategies are formulated and compiled into a list on an easily accessible card, including individual and social strategies to distract from suicidal crises and to ensure a safe environment and phone numbers for easy access to relevant care services, helplines, and supportive friends/relatives as chosen by the participant. A fourth session is optional, when there is a need for repetition and practicing of the strategies. A 2-year outreach component follows, during which participants are sent semi-standardised letters (signed by the therapist) to help them remember to use the strategies learned during the ASSIP sessions and to let them know that they can contact the therapist should need arise, and to provide easy access to health care system. If a new suicidal crisis emerges, the ASSIP therapist offers a “booster” session to go through the case conceptualisation again and the safety plan is updated.

There is a real need for fully powered studies of brief preventive interventions for older adults after non-fatal suicidal acts and older adults hospitalised for suicidal prevention. Drawing from the promising results of ASSIP in adults we will now conduct a randomised controlled trial (RCT) to evaluate a modification of ASSIP specifically designed to meet the needs of suicidal persons aged 65 and above, the ASSIP-Older Adult program (ASSIP-OA).

## Objectives and hypothesis

This paper outlines the study protocol for our upcoming study, which aims to evaluate a modified version of the Attempted Suicide Short Intervention Program (ASSIP), specifically for older adults (65+), the ASSIP for older adults (ASSIP-OA), as an add-on to usual treatment. The efficacy of the ASSIP-OA intervention will be evaluated by comparing it to a control group receiving only treatment as usual (TAU).

The main hypothesis is that participants randomised to the add-on treatment condition - the ASSIP-OA intervention - will experience a preventive effect on suicide and suicide attempts within 6, 12, and 24 months following the index episode. Also, we anticipate participants in the experimental condition to show better improvement in psychiatric symptoms/severity of suicidal ideation/coping skills/quality of life (secondary outcomes). Additionally, we aim to investigate if the brief preventive intervention (ASSIP-OA) is feasible, based on its acceptability, appropriateness, deliverability and fidelity, and whether participants have fewer days of inpatient care or experience any negative side effects. Further, the study also includes a qualitative approach to explore what kind of challenges and opportunities (a) older adults and (b) ASSIP-OA therapists experience in relation to implementing the brief preventive intervention, and if the modifications that we made in ASSIP-OA are relevant for older adults.

## Methods

### Study setting and design

This study is designed as a multicentre two-group parallel randomised controlled trial (RCT), to be conducted within psychiatric services in the Swedish regions of Västra Götaland, Skåne, and Halland. Several additional regions have shown interest and may be added. Participants are randomly assigned to either:

- a brief 3–5 session preventive intervention (approx. 1 session per week), the Attempted Suicide Short Intervention Program for Older Adults (ASSIP-OA), which is an add-on to usual treatment.
- a control group receiving treatment as usual (TAU) only.

**Fig. 1:**
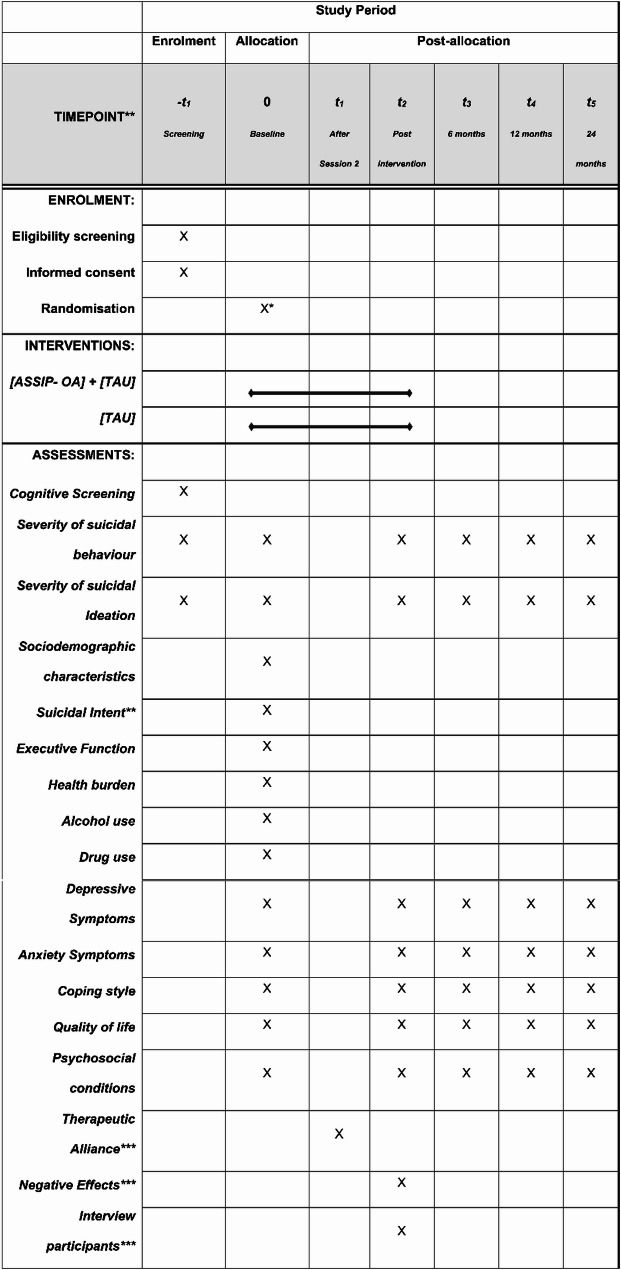
SPIRIT flow diagram. Schedule of enrolment, interventions, and assessments. * Participants are randomised after completion of the baseline assessment. **Suicide attempters only. *** Participants allocated to ASSIP-OA only

Measurements will be taken prior to randomisation and after the last session (or after 5 weeks for the control group) and follow-up measurements (see below) will be assessed at 6, 12 and 24 months after the last session. An overview of data to be collected at the various timepoints is shown in Fig. 1. Following the baseline measurement, randomisation will take place electronically via REDCap (Research Electronic Data Capture) [28] and will be conducted using block randomisation with varying block sizes and with an allocation ratio of 1:1 between intervention groups. The randomisation procedure was designed by a statistician at the University of Gothenburg, independent of the research group. Randomisation will be blinded to the data processors, as well as to the researchers and clinicians conducting the initial assessments and screening (prior to randomisation). The size of the randomisation blocks will remain undisclosed. Due to the design of the intervention, blinding participants to their assigned group will not be possible. As it is anticipated that usual treatment will vary somewhat between the study locations, it will be mapped for each patient. Data on clinical diagnoses, healthcare contacts and treatments for mental health concerns during 24 months before and after baseline measurements are derived from medical records.

The study will follow the CONSORT guidelines and was pre-registered at Clinical Trial.gov NCT06831942 on February 21, 2025 and revised May 21, 2025. The present study protocol follows the recommendations for clinical trial protocols from the SPIRIT Declaration [29, 30], see Supplementary material 1 for checklist.

### Participants and recruitment

Participants are recruited from psychiatric healthcare settings at participating centres, including inpatient psychiatric wards, outpatient psychiatric services, emergency care, and medical wards via consultation liaison services. Patients (65+) who have been hospitalised (minimum 1 night) in connection with a suicide attempt during the last 3 months or an episode of serious suicidal planning during the last month at any of the participating clinics are eligible for participation in the study. Unit managers, doctors and medical staff working in the inpatient wards, outpatient clinics and consultation psychiatric services will be informed about the study and asked to identify potential participants and provide interested patients with oral and written information about the trial.

Individuals who meet the eligibility criteria (see below) are invited to a brief screening to confirm their suitability for the study. If they remain eligible and wish to receive more information, the staff will provide detailed study information, which participants can review at their own pace. They will also have the opportunity to ask questions, which can be addressed by the study researcher as needed. The study researcher will then contact the patient to discuss the research details and answer any remaining questions. If the patient chooses to enrol, they will sign the consent form, and baseline measurements will begin. After the baseline assessment, the participant is randomised to either the intervention group (ASSIP OA + TAU) or the control group (TAU). Participants receive information about allocation in connection with the baseline visit, along with information about their scheduled appointment with an ASSIP-OA therapist or their next scheduled research assessment.

All quantitative data will be securely stored in REDCap and presented at the group level to ensure anonymity.

### Eligibility criteria

#### Inclusion criteria

- Age 65 and above at time of index suicide attempt or hospitalisation for serious suicidal plans.
- A mental health care contact during the active treatment period.
- Capable of understanding study procedures and providing informed consent.

#### Exclusion criteria

- Clinical diagnosis of dementia or Montreal Cognitive Assessment (MoCa) score less than − 2 standard deviations from the normative score for education and age [31]. Ongoing delirium, or any other condition impeding the comprehension of the study’s procedures and implications that hinder the provision of informed consent.
- Severe ongoing psychosis, severe ongoing substance use disorder, emotionally instable personality syndrome and any other condition that would require longer specialized treatment to reduce future suicidal behaviour (e.g. DBT).
- Terminal illness.
- Insufficient knowledge of the Swedish language (requires interpreter).
- Aphasia or other severe communication issue or severe hearing and/or severe visual impairment despite corrective aids that render the intervention unfeasible.

In connection with post-treatment data collection, a purposive sample of approximately 15 participants who took part in ASSIP-OA sessions will be asked to participate in an interview to generate a deeper picture of participants’ experiences of ASSIP-OA. In addition, we will use focus group interviews to examine ASSIP-OA therapists’ experiences of the training and application of ASSIP-OA in their clinical settings, including their experiences of obstacles and opportunities in connection with the delivery of ASSIP-OA. We anticipate that all the ASSIP-OA therapists (n = approx. 14) will be interested in taking part in two sets of focus groups, but participation is voluntary.

### Sample size

A total of 132 participants will be included in the trial. The power calculation, i.e., the number of participants needed in each intervention group, is based on the previous ASSIP study of Gysin-Maillart and colleagues [17]. In that study, 26.7% of the participants in the control group, usual treatment, and 8.3% in their intervention group, ASSIP, reattempted suicide during follow-up. Based on clinical experience as well as prospective data from our previous multicentre study [12] for persons 65 + receiving usual treatment, we choose to calculate power based on an expected repetition risk of 30% in our control group (usual treatment), and 10% in our intervention group receiving ASSIP-OA plus TAU. Furthermore, given a desired power of 0.80 (a 4/5 chance of discovering an effect if it exists, regarded as adequate in this context) and a risk level of 5%, 60 participants in each condition is needed. We further expect a dropout rate of 10%, thus each group will therefore comprise 66 participants.

### Therapists

The therapists (psychiatrists, psychologists, nurses in psychiatric care, or social workers) all work as mental health professionals at the participating clinics, and all have clinical experience with suicidal patients. Presumptive ASSIP-OA therapists are required to have basic education in psychotherapy. The training to become a certified ASSIP therapist includes two days of theoretical lectures and skills training followed by supervision for approximately one year (5 patients under supervision, supervision on every session). The ASSIP-OA therapist training also includes theoretical lectures about suicidal behaviour in older adults as well as information about how the intervention has been modified to apply to older participants. The ASSIP-OA therapists are trained by a certified ASSIP supervisor (A.E.), as well as clinical gerontologists experienced in geriatric psychiatry. Only fully trained and certified ASSIP therapists or therapists in training with supervision after each session will conduct ASSIP-OA sessions with participants in this study. During the course of the project, the ASSIP-OA therapists have group meetings and continued regular supervision in groups or individually even after certification.

### Interventions

#### Treatment group

The treatment group will receive TAU with the addition of a modified versionof ASSIP. The full treatment protocol for ASSIP-OA is described in Table 1. The novelty of this program for older adults and the main modifications include:

- Flexibility in treatment delivery, such as offering home visits, breaks and shorter/longer sessions as needed.
- Optional additional session together with one or two close relatives or other support person(s), chosen by the participant.
- Optional additional session to further train safety strategies (may be needed more frequently for participants with MCI or other disabilities).
- Homework, letters, and case conceptualization adapted linguistically and content-wise to the target group.
- ASSIP-OA also includes participants with a suicidal crisis (e.g. suicide plans/thoughts requiring hospital care).

Clinical psychologists and a psychiatrist, experienced in geriatric psychiatry in Sweden, have drawn on insights from the geriatric psychiatry clinic in Bern (personal communication, Anja Gysin-Maillart, 2025) to tailor the treatment protocol to the Swedish context. Gysin-Maillart stresses that it is not the specific components of ASSIP that require change, but rather that there should be a greater focus on the patient’s individual needs of support in areas such as loneliness, chronic pain, physical limitations and in some cases milder cognitive impairment. Gysin-Maillart highlights the need for a higher degree of flexibility when delivering ASSIP to older adults, especially for the “older older”. Providing home visits is one way to improve adherence [32]. Older adults may require extra support in communicating their needs. As most suicidal older adults are dealing with chronic medical conditions and have regular appointments with primary care, it is important to share the case conceptualisation with the patient’s GP and other relevant care providers, once permission is given by the patient. Moving to long term care was a particularly vulnerable time for older adults at the Bern clinic, an observation we have also made in our own Swedish research [33]. In such cases, ASSIP could better fit the older person’s needs by including a session with a nurse or other staff member at the care facility, chosen by the older adult, after the tree core sessions to support the translation of ASSIP warning signs and coping strategies into everyday life. Another reflection from the Bern clinical experience was that, while the video playback was not an issue for most older participants, some older men found it problematic to watch. This could be ameliorated by the therapist taking a more supportive role than they might have done with a younger patient. A more active role by the therapist may also be appropriate for older adults experiencing cognitive impairment. Enhancing problem-solving abilities is a key component of treatment protocols for individuals with suicidal tendencies and executive dysfunction [34].

**Table 1:** Treatment protocol for the attempted suicide short intervention program for older adults (ASSIP-OA)

Session no	Session Components and Modifications
1	**Narrative interview.** Narrative interview focusing on the background of the suicidal crisis. At the end of session 1 the patient receives a psychoeducational text to read and comment on as homework. The homework has been modified by psychologists (S.H, A.I.B and S.W) with experience in geriatric psychiatry together with a certified ASSIP instructor and supervisor (A.E). In addition, an older adult (80+) with lived experience of attempted suicide has contributed to the revision of the homework.
2	**Video playback.** Starts with collaborative with the patient’s comments in the homework text. The patient and therapist then watch the video recording from session 1 together, periodically pausing the video to comment and add information.
3	**Collaborative case conceptualisation.** Compilation of a written case conceptualisation of the individual’s vulnerability, triggering events, and suicidal process that preceded the suicidal crisis. Long-term therapy goals, warnings signs, and safety strategies revealed and developed (during video-playback) by the patient and the therapist collaboratively are revised and compiled into a list on an easily accessible card, including phone numbers to easy access relevant care services, helplines, and people whom the patient chooses and trusts. A copy of the case conceptualization, including safety measures, and long-term goals is sent to the patient’s physician as well as to other relevant care providers. The ASSIP-OA intervention may end here, or further sessions may be relevant as described below.
4 (optional)	**Rehearsal session.** This session, which is optional also in the original ASSIP program, may be particularly relevant for older adults with mild cognitive impairment or other functional limitations who may require an extra session to further train safety strategies.
5 (optional)	**Session including support person.** This session (a modification not included in the original ASSIP) invites one or two close relatives or other support person(s), chosen by the participant. The patient shares his/her formulation of preventive measures, and a dialog follows, with a focus on how safety measures learned in ASSIP-OA can be translated into everyday life.
Letters	**Letters.** After completion the last ASSIP-OA session, therapists will send semi-standardised letters every third month during the first year and biannually during the second year. The purpose of these letters is to provide hope and psychological support, as well as providing participants with a gentle reminder to make use of their personalised coping strategies. The patient is encouraged to contact their ASSIP-OA therapist, either to update them on their well-being or if they experience a new suicidal crisis. In the latter case, the participant’s physician is informed, and the ASSIP-OA therapist can offer a booster session for additional support facilitating access to the healthcare system as needed, with a renewed conceptualisation and updated formulation on safety measures. The session can be provided in hospital as needed. A copy of the updated materials is sent to the participant’s physician.
Non session specific modifications in ASSIP-OA	A higher flexibility when delivering ASSIP-OA, providing the opportunity for home delivered session if needed. Depending on physical and cognitive functioning, flexibility in session length and breaks is advisable. A more active role on the part of the therapist could also be relevant for older adults who suffer from mild cognitive impairment. Linguistically and content-wise modifications to suite the target group, adjusted homework, letters, and case conceptualisation.

#### Control group

Participants randomised to the control group will receive TAU in line with the regional and national guidelines. Treatment as usual (TAU) was selected as the comparator to reflect the standard care typically provided to suicidal older adults in real-world clinical settings. TAU may vary between the participating hospitals. The cornerstone of treatment as usual for suicidal older adults consists of antidepressant medication, alone or in combination with other types of psychoactive drugs (sedatives/antipsychotics/hypnotics/mood stabilisers). Persons with serious or treatment-resistant depression may be offered electroconvulsive therapy (ECT). Some persons may receive supportive/therapeutic contacts with mental health professionals (nurses/social workers/psychologists) but no psychosocial interventions designed specifically for suicidal older adults are offered as part of ordinary treatment at the participating clinics.

### Screening

Potential participants receive written information about the research project prior to screening. The screening begins with information about what it means to be part of the study. In order to determine eligibility, the short form of the Columbia Suicide Severity Rating Scale (C-SSRS) [35] is employed to investigate suicidal planning and behaviour. The Montreal Cognitive Assessment (MoCA) [36] is applied for cognitive screening. Severe ongoing psychosis, severe ongoing substance use disorder and emotionally instable personality syndrome are screened through the patients’ medical record. The screening procedure takes place at the patient’s ward/outpatient clinic.

### Sociodemographic and clinical characteristics

To characterise participants, data on demographic variables including age, sex (woman, man, non-binary or other), marital status, ethnicity, education level, and occupation prior to retirement will be gathered after study inclusion.

The Trail Making Test (TMT part A and B) [37] will be employed to assess executive function. The full (original) version of the Columbia-Suicide-Severity-Rating Scale (C-SSRS) [35] will be administered by the interviewer for history of suicidal episodes and to provide information for a global clinical risk assessment (the short form of the C-SSRS has been administered at the screening, see Fig. 1). For actual suicide attempts, suicidal intent is measured with Beck’s Suicide Intent Scale (SIS) [38]. Overall health burden will be rated with the Cumulative Illness Rating Scale for Geriatrics (CIRS-G) [39]. Alcohol use will be self-reported at this time with the Alcohol Use Disorders Identification Test (AUDIT) [40], and persons who acknowledge drug use will complete the Drug Use Disorders Identification Test (DUDIT) [41].

### Outcome measures

#### Primary outcome

The primary outcome variable at 6-, 12- and 24-month follow-up is any “new suicidal episode,” a composite rating that is coded “yes” in the event of (a) a new suicide attempt as registered in the participant’s medical record or the national hospital register or (b) suicide death as registered in the national cause of death register.

#### Secondary outcomes

The following secondary effect measures will be collected at baseline, immediately after the intervention (for the control group 5 weeks after baseline) as well at the 6-, 12- and 24-month follow-ups.

##### *Severity of suicidal ideation*

Severity of suicidal ideation will be rated with the Columbia-Suicide Severity Rating Scale (C-SSRS) [35]. Scores range from 0 to 5, where higher scores indicate more severe ideation.

##### *Depressive symptoms*

To assess depressive symptoms the Montgomery-Åsberg Depression Rating Scale (MADRS) [42], will be applied. Each item yields a score of 0 to 6; the overall score thus ranges from 0 to 60. Higher MADRS score indicates more severe depression.

##### *Anxiety symptoms*

Generalized Anxiety Disorder 7-item scale (GAD-7) [43] will be used to assess anxiety symptoms. Each item yields a score of 0 to 3; the overall score thus ranges from 0 to 21. Higher GAD-7 score indicates more severely elevated anxiety.

##### *Coping style*

Coping styles will be assessed by Brief - Coping Orientation to Problems Experienced Inventory (Brief-COPE) [44]. The 28-item self-report scale is scored from 1 to 4 where 1 = I haven’t been doing this at all 2 = A little bit 3 = A medium amount 4 = I’ve been doing this a lot. The Brief COPE consists of 14 subscales, each representing a specific coping strategy. Each subscale consists of 2 items, making the full scale 28 items in total. Scores are presented as average scores (sum of item scores divided by number of items), indicating the degree to which the respondent has been engaging in that coping style.

##### *Quality of life*

Health-related quality of life is rated with the The EuroQol 5-Dimension 5-Level (EQ-5D-5 L) [45]. The EQ-5D-5 L consists of the EQ-5D descriptive system and the EQ visual analogue scale (EQ VAS). The descriptive system comprises five dimensions (mobility, self-care, usual activities, pain/discomfort and anxiety/depression): Each dimension has 5 levels: A higher 1-digit number mean a worse outcome. Higher scores on the EQ VAS mean a better outcome.

##### *Psychosocial conditions*

In addition, participants will fill in a brief psychosocial questionnaire with 6 single item questions inspired by scales employed in previous studies focusing on older adults’ mental health [46, 47, 48, 12]. Items are scored on a four-point Likert-scale (0–3). “Do you feel that you matter to others?” “Have you felt like a burden to others recently?” “Do you believe others would be better off without you?” “Have you had problems with loneliness recently?” “Do you feel that your situation has been hopeless recently?” “Have you had difficulties in your relationship with a partner, family member, or another important person recently?”

### Therapeutic alliance, health-care utilization, negative effects of treatment and feasibility measures

#### Therapeutic alliance

The Working Alliance Inventory-Short Revised (WAI-SR) [49], is used to measures therapeutic alliance on 12 items on a 7-point Likert scale from 1 = “never” to 7 = “always”, after the second ASSIP-OA session.

#### Health-care utilisation

We will assess psychiatric inpatient hospitalizations, as well as the number and type of psychiatric healthcare contacts, based on medical records at 12- and 24-month follow-ups.

#### Negative effects

Further, the Negative Effects Questionnaire (NEQ) [50], will be applied after the last ASSIP-OA session to answer the research question on side effects. NEQ contains of 32 items that are scored on a five-point Likert-scale (0–4) and differentiates between negative effects that are attributed to treatment and those possibly caused by other circumstances. At the end of the questionnaire there is also one open-ended question: “Describe in your own words if any other negative events or effects occurred, and what characterised them.”

#### Feasibility measures

The following feasibility measures is used to investigate if the modified ASSIP intervention is feasible in older adults: Acceptability will be addressed in the qualitative interviews with participants post intervention. Appropriateness is measured by Cronbach alpha and mean inter-item correlation in our questionnaires (see above under effect measures). Deliverability is measured by examining that all components of the ASSIP- OA are provided on the 3–5 sessions without having to stress through them. Video recordings will be used to explore this. Fidelity: To ensure good administration of the ASSIP-OA: (1) training manuals with checklists for the therapists will be used to check after each session, and (2) video recordings of randomly selected ASSIP- OA sessions (15%) are reviewed by independent assessors using a checklist to rate the therapist’s competence and adherence to the intervention (Adherence and Competence Scale (ACS)– Gysin-Maillart et al., soon to be submitted).

### Retention

Participants will always receive a scheduled appointment for an ASSIP-OA session in writing, either by mail, text message, or directly handed to them. The research team will remind participants of scheduled times to administer questionnaires by phone. Each participating centre will have a designated contact person available to address questions about the study or the recruitment process. Additionally, the research team will make regular visits to participating centres to provide support, answer questions, and reinforce awareness of the trial among healthcare staff.

### Data management

All quantitative data will be managed by Research electronic data capture (REDCap) an electronic software solution [28] that will be used for data collection and randomisation in the planned study. As REDCap is used for data monitoring, and given that this is a very brief intervention, consistent with other ASSIP trials, it does not warrant extensive oversight, and a Data Monitoring Committee (DMC) is thus not required. Qualitative data will be audio-recorded and transcribed in by a professional. All data will be kept in in fire-safe locked cabinets at the research unit to ensure participant confidentiality, and the code key will be stored separately. All data will be presented at group level so that no individual can be identified. All members of the research theme are educated and will adhere to the principes of Good Clinical Practice (GCP).

### Data analysis

We will use an intention-to-treat analysis. The primary outcome will be analysed using chi-square analyses. The secondary outcomes will be analysed using a so-called “mixed effect model approach” with the aim of examining changes before and after treatment as well as during the follow-ups. This approach can take into account data clustering and covariates as well as possible dropouts.

We will examine effects both between and within groups, to be able to determine if the preventive intervention ASSIP-OA in addition to TAU has a better effect than the control group, treatment as usual (TAU), after the intervention (between-group effect). Further, we want to find out whether ASSIP-OA and the control group have improved over time (within-group effect) and, if the ASSIP-OA has any effect, whether such effect is “driven” by the ASSIP-OA (interaction effect). The statistical approach is based on regression analyses. Contrary to more traditional variable-oriented analyses for repeated measurements, it has the benefit of taking into consideration the fact that data are not independent at repeated measurements over time and provides better handling of missing data by incorporating all available information. This method is now regarded as preferable to using more traditional methods for missing data in clinical studies.

For qualitative data collected through individual and focus group interviews, transcribed interviews will be analysed, and themes and subthemes will be identified and categorised following Braun and Clarke’s thematic analysis [51].

### Ethics

Ethical approval for the planned trial was granted by the Swedish Ethical Review Authority (2024-07392-01). The study therapists will continuously evaluate the participants’ suicidal ideation/planning at each treatment session. Any indication of increased suicidal ideation at any point during the project will prompt immediate contact with the participant’s care professionals. The participant’s physician will be informed without delay if a clinically relevant rise in suicidal ideation or other signs of acute distress are noted during the study. Adverse events will be registered at each session. Participation will be entirely voluntary, with no financial or other incentives, and all participants must provide written informed consent. In line with our ethical approval, patient insurance coverage is ensured and available to participants should the need arise.

## Discussion

Suicide rates remain high among our oldest citizens, but older adults are still gravely underrepresented in clinical suicide prevention research [52]. It is our hope that the development and evaluation of ASSIP-OA will help to mitigate this gap.

This multicentre preventive intervention study, conducted as an RCT, aims to evaluate a modified version of the ASSIP intervention as an add on to TAU specifically for older adults (65+). A major adaptation is that we will include also persons with a suicidal crisis requiring hospital care, as most older adults do not survive their first suicide attempt. Novel components of the older adult treatment program include; flexibility in treatment delivery, optional session together with one or two close relatives or other support person(s), and homework, letters, and case conceptualisation are adapted linguistically and content-wise to the target group. Additionally, we aim to investigate if the brief preventive intervention (ASSIP-OA) is feasible, based on its acceptability, appropriateness, deliverability and fidelity, and if participants experience any negative side effects. Further, in interviews with participants and focus groups with therapists we will identify challenges and opportunities that may arise in the implementation of a brief preventive intervention like ASSIP-OA.

Despite careful planning, the study has some limitations. First, we recognise that participant recruitment may be challenging. To address this, we have collaborated with participating centres to facilitate recruitment by providing training for therapists and medical staff responsible for screening, as well as engaging key contact persons who can monitor referrals to clinics and wards. Additionally, recruitment materials and contact information have been distributed to streamline the process. Second, the Swedish healthcare system differs from the context in which ASSIP was initially developed and evaluated. Access to psychotherapy in Sweden is much more limited, and almost non-existing for older adults, restricting opportunities to pursue long-term therapeutic goals identified by the intervention. To mitigate this discrepancy, we include only participants who have a designated healthcare physician responsible for their mental health. We also make efforts to contact the physician before and after the preventive intervention, sharing the case conceptualisation and advocating for its integration into the patient’s ongoing care plan.

If this suicide prevention intervention trial yields positive outcomes and proves feasible, the results could inform the development and implementation of a more person-centred care for suicidal older adults.

### Trial status

Protocol version 1 Registered Feb. 21, 2025, revised May 21, 2025. Recruitment is scheduled to begin in June 2025, and preliminary date for recruitment completion is 30 June, 2029. If there will be any important protocol modification, this will render an additional application to the Swedish ethical approval board and a modification of the trial registration.

## Electronic supplementary material

Below is the link to the electronic supplementary material.

Supplementary Material 1

## Data Availability

No datasets were generated or analysed during the current study.

## Declarations

### Roles and responsibilities

The funders had no role in the study design; data collection, management, analysis, or interpretation; or in the writing or publication of the report. Region Västra Götaland is the trial sponsor, responsible for overall management of the trial, including ethical oversight and trial conduct.

## Abbreviations

ASSIP: Attempted Suicide Short Intervention Program
ASSIP-OA: Attempted Suicide Short Intervention Program for Older Adults
AUDIT: Alcohol Use Disorders Identification Test
Brief-COPE: Brief - Coping Orientation to Problems Experienced Inventory
CIRS-G: Cumulative Illness Rating Scale for Geriatrics
CONSORT: Consolidated Standards of Reporting Trials
C-SSRS: Columbia-Suicide Severity Rating Scale
DUDIT: Drug Use Disorders Identification Test
EQ-5D-5 L: EuroQol 5-Dimension 5-Level
GAD-7: Generalized Anxiety Disorder 7-item
GCP: Good Clinical Practice
MADRS-S: Montgomery-Åsberg Depression Rating Scale - Self report
MoCa: Montreal Cognitive Assessment
NEQ: Negative Effects Questionnaire
RCT: Randomised Controlled Trial
REDCap: Research Electronic Data Capture
SIS: Suicide Intent Scale
SPIRIT: Standard Protocol Items: Recommendations for Interventional Trials
TAU: Treatment As Usual
TMT: Trail Making Test
WAI-SR: Working Alliance Inventory - Short Revised
